# Possible involvement of interleukin-18 in the pathology of hepatobiliary adverse effects related to treatment with ceritinib

**DOI:** 10.1186/s12885-018-4913-5

**Published:** 2018-10-19

**Authors:** Taizou Hirano, Akira koarai, Tomohiro Ichikawa, Teruyuki Sato, Takashi Ohe, Masakazu Ichinose

**Affiliations:** Department of Respiratory Medicine, Tohoku University Graduate School of Medicine, 1-1 Seiryou-machi, Aoba-ku, Sendai, 980-8574 Japan

**Keywords:** Lung cancer, Ceritinib, Drug induced hepatobiliary adverse events, Interleukin 18

## Abstract

**Background:**

Ceritinib demonstrated a statistically significant effect on the progression-free survival versus chemotherapy in patients with advanced anaplastic lymphoma kinase (ALK) rearrangement in non-small cell lung cancer (NSCLC) as the first therapy or after previous treatment with crizotinib and one or two prior chemotherapy regimens in global phase 3 studies. However, some serious adverse effects related to ceritinib therapy were reported across these clinical studies. Among them, a grade 3 and 4 increase in hepatobiliary enzymes was one of the common adverse events related to treatment with ceritinib. However, the pathology remains unclear. Previously, increased Interleukin (IL)-18 was observed in both biliary duct disease and liver disease. Therefore, we hypothesized that IL-18 is involved in the pathology of hepatobiliary adverse effects related to treatment with ceritinib and evaluated the serum IL-18.

**Case presentation:**

The patient was a 53-year-old Japanese woman that we previously reported as having severe hepatobiliary adverse effects related to ceritinib therapy. Laboratory data, CT and MRI were obtained at each time point. IL-18 was evaluated by ELISA method at each time point. Immunochemical staining of liver tissue was performed as a standard protocol using antibodies against IL-18. Our records showed that the levels of serum IL-18 increased from the early stage of hepatobiliary adverse effects related to the treatment with ceritinib and were became worse with an increase in hepatobiliary enzymes and the progression of imaging abnormalities in the bile duct. Furthermore, IL-18 positive cells were detected in the inflammatory sites around the interlobular bile duct of the liver tissue.

**Conclusion:**

Our case report shows that the increase of serum IL-18 had a positive correlation with the progression of severe hepatobiliary adverse effects related to treatment with ceritinib and the involvement of IL-18 in the hepatobiliary inflammation by pathological evaluation. These results suggest that IL-18 could be a useful surrogate marker for the hepatobiliary toxicity of ceritinib. However, this is only one case report and further prospective observations will complement our data in the future.

## Background

Anaplastic lymphoma kinase (ALK) rearrangement occurs in approximately 2–7% of patients with non-small cell lung cancer (NSCLC). Ceritinib is a new generation selective tyrosine kinase inhibitor of ALK that provided longer progression-free survival than chemotherapy in NSCLC patients harboring an anaplastic lymphoma receptor tyrosine kinase gene rearrangement when used as the first line therapy in a randomized global phase 3 trial [1]. However, the data of the phase 1 to 3 studies in patients with advanced ALK-positive NSCLC have shown that an increase in hepatobiliary enzymes is one of the common adverse events related to treatment with ceritinib, including serious cases (Grade 3 and 4) [1–5]. Moreover, we previously reported that ceritinib could be a potential cause of severe hepatobiliary adverse effects including drug-induced cholestasis with radiologically abnormal findings of the biliary tract even after its discontinuation [6]. However, the pathology of hepatobiliary adverse effects related to treatment with ceritinib remains unclear. Previously, increased interleukin (IL) 18 was observed in both biliary duct disease and liver disease, and a relationship with the pathology was reported [7, 8]. Therefore, we hypothesized that IL-18 is involved in the pathology of hepatobiliary adverse effects related to treatment with ceritinb and evaluated the serum IL-18.

## Methods

The patient was a 53-year-old Japanese woman that we previously reported as having severe hepatobiliary adverse effects related to the treatment with ceritinib including drug-induced cholestasis with radiologically abnormal findings of the biliary tract [6]. Laboratory data, computerized tomography (CT) and magnetic resonance imaging (MRI: T2, TSE) were obtained at each time point. The level of IL-18 and IL-6 was measured by ELISA method (LSI Medicine Corporation, Japan, SRL Corporation, Japan. respectively) at each time point. Liver tissue of the patient was obtained two months after the treatment. Immunochemical staining of the liver tissue was performed as previously described using antibodies against IL-18 (SC-133127, SantaCruz) [9].

## Case presentation

**Fig. 1:**
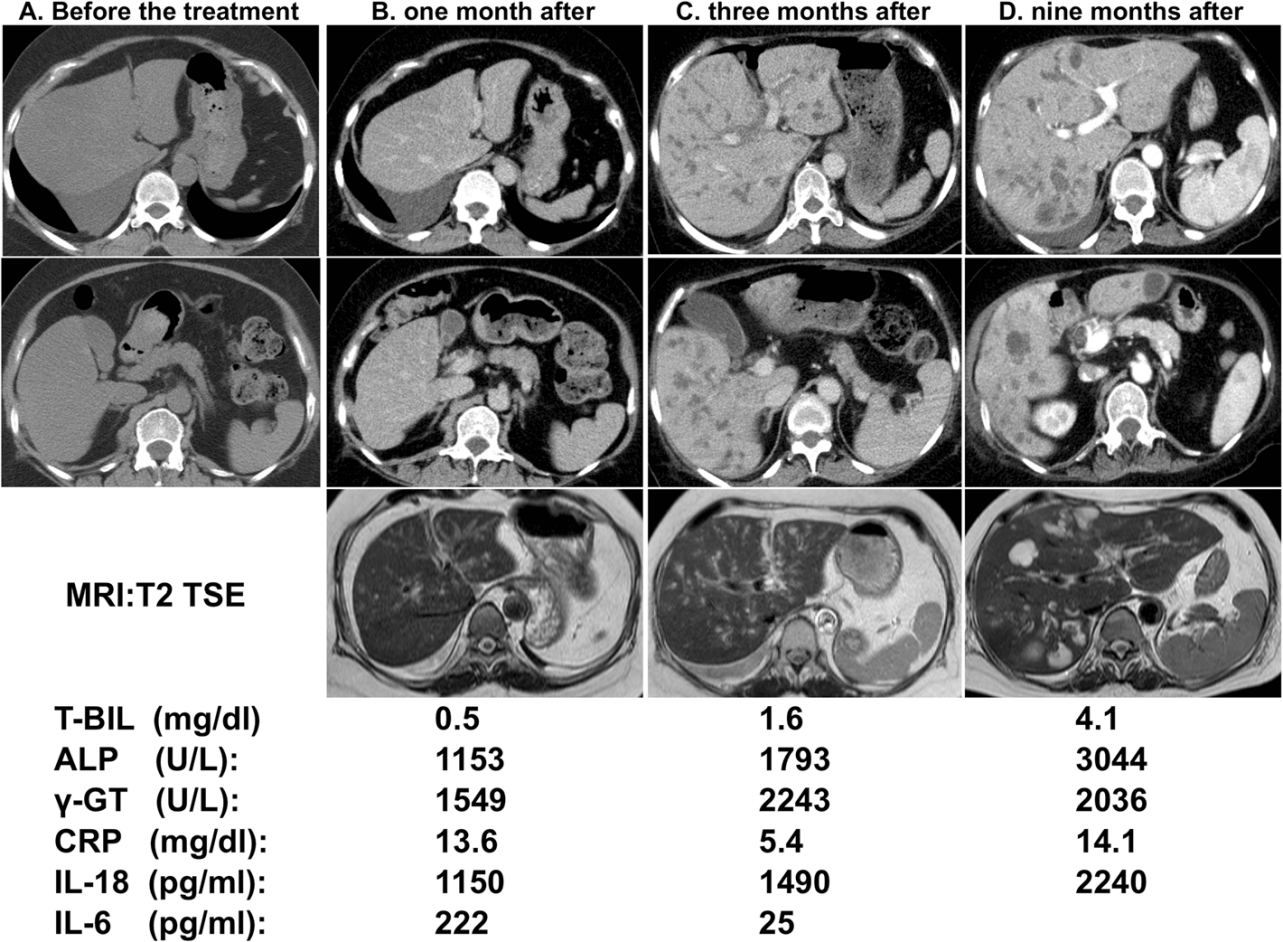
Computer tomography (CT), magnetic resonance imaging (MRI, T2 TSE) and laboratory results during the clinical course. **a**-**d** Abdominal CT and MRI before **a**, one month **b**, three months (**c**) and nine months (**d**) after treatment with ceritinib. **a**, **b** Abdominal CT and MRI showed no abnormalities of the liver. **c** CT showed a dilation of the intrahepatic bile duct and MRI also showed dilation of the intrahepatic bile duct with periportal edema. **d** CT and MRI showed a dilation of the intrahepatic bile duct and newly detected biloma. The results of serum T-Biil, ALP, γGT, CRP, IL-18 and IL-6 in the clinical course are shown under the imaging study. Serum IL-6 was decreased, but serum IL-18 was increased with the progression of the ceritinib-induced DIC even after its discontinuation and treatment with PSL

**Fig. 2:**
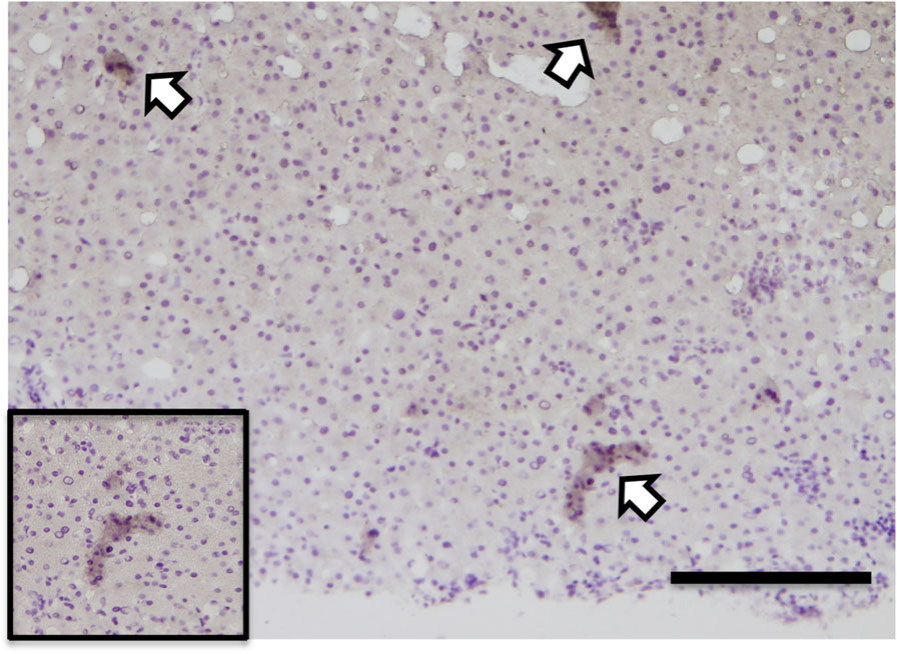
Representative photographs of immunocytochemical staining against IL-18. A portion of the liver tissue from the liver biopsy was also immunostained with antibody to IL-18 (brown; arrows), and the nuclei were identified with hematoxylin-eosin stain (× 100). Bar, 200 μm. In the window, a high magnification image (× 200) is shown. IL-18 positive cells were detected in the inflammatory sites around the interlobular bile duct of the liver tissue

A 53-year-old Japanese woman was diagnosed with lung adenocarcinoma (pT1aN0M0, Stage IA) harboring an ALK rearrangement and had been administered crizotinib after postoperative recurrence. She had a history of diabetes mellitus. Follow-up CT revealed mediastinal lymph node metastasis indicating tumor progression and then ceritinib was started. One month after ceritinib treatment, fever, an increase in the serum IL-18 level, inflammatory markers (CRP and IL-6) and bile tract enzymes (ALP and γ-GT) was observed (Fig. 1a, b). To evaluate the cause of the elevated bile tract enzymes, we next performed a liver biopsy and the patient was diagnosed as ceritinib-induced cholestasis from the pathological examination of her liver tissue. Then, ceritinib was discontinued and we also started treatment with prednisolone to attenuate the inflammatory responses in the bile duct caused by ceritinib. However, though fever and the serum levels of CRP and IL-6 decreased with the treatment, the serum levels of IL-18 and hepatobiliary enzymes increased together with the expansion of the intrahepatic bile duct on CT and MRI (Fig. 1c) even two months after the discontinuation of ceritinib. After 9 months, the serum IL-18 had increased more together with the progression of the bile duct dilation and the appearance of biloma on CT and MRI (Fig. 1d). To determine the source of serum IL-18, we evaluated the expression of IL-18 in the liver by immunostaining. IL-18 positive cells were detected in the inflammatory sites around the interlobular bile duct of the liver tissue (Fig. 2).

## Discussion and conclusions

Ceritinib demonstrated a statistically significant effect on the progression-free survival versus chemotherapy in patients with advanced ALK rearrangement NSCLC as the first line treatment or after previous treatment with crizotinib and one or two prior chemotherapy regimens in global randomized phase 2 and 3 trials [1, 5]. However, in these clinical studies, some serious adverse effects related to ceritinib therapy such as gastrointestinal symptoms (nausea, vomiting and diarrhea) and abnormal laboratory data were reported. In fact, in the ASCEND 4 and 5 studies, serious adverse effects were reported in 15.9% and 11.3% of patents, respectively [10]. Among them, an increase in hepatobiliary enzymes was one of the common adverse effects related to treatment with ceritinib. In the ASCEND-4 study, grade 3 and 4 increases in ALT, AST and γ-GT occurred in 31%, 17% and 29% of patients, respectively [1]. Similarly, in the ASCEND-5 study, grade 3 and 4 increases in ALP were reported in 6% of the patients [5]. Furthermore, in the ASCEND-1 and 5 studies, 1.6 and 1.7% patients discontinued ceritinib because of hepatobiliary adverse effects (hepatitis cholestatic (*n* = 1), increased blood alkaline phosphatase (*n* = 1), increased alanine transaminase (*n* = 2) and increased aspartate transaminase (*n* = 2)) [2, 5]. In this report, our records showed that the levels of serum IL-18 increased from the early stage of severe hepatobiliary adverse effects, including drug-induced cholestasis with radiologically abnormal findings of the biliary tract and were becoming worse with the increase in the hepatobiliary enzymes and the progression of imaging abnormalities in the bile duct. These results suggest that IL-18 was related to the pathology and could be a useful marker for the state of the disease in hepatobiliary adverse effects related to treatment with ceritinib. IL-18, which is mainly produced by Kupffer cells and macrophages, plays a major role in immunoregulation and inflammation. In human, IL-18 has a role in several autoimmune and inflammatory diseases including liver, and an elevation of serum IL-18 has been also reported in biliary duct disease such as primary biliary cirrhosis and was correlated with the levels of serum bilirubin, in which IL-18 is involved in the cellular immune response to the bile duct via activation of the Th1 pathway [8, 11].

Some limitations and caveats should be mentioned with respect to this case. First, a previous report showed that the serum IL-18 levels of patients with lung cancer were significantly higher compared with normal subjects (the average value from the serum IL-18 of normal subjects was 227.3 pg/ml vs that of patients with lung cancer, which was 390.9 pg/ml) and those of the NSCLC patients with stage IV were significantly higher than those in patients with stage IIIb [12]. In our case, although we did not evaluate the baseline level of IL-18 before both introducing ceritinib and crizotinib, we still believe that the value of IL-18 in our case was clinically important value because it was definitely higher than those previously reported lung cancer patients. Second, it remains unclear from our case report whether there is a direct relationship between the increased level of IL-18 and ceritinib treatment. A previous report showed that increased levels of serum IL-18 were observed in both biliary duct disease and liver disease and the increase in serum IL-18 had a positive correlation with the severity of liver disease [7, 8, 13]. From these reports, the increased level of serum IL-18 in our case report may indicate hepatobiliary toxicity rather than ceritinib-related hepatobiliary toxicity.

In conclusion, the findings of our case indicated that the increase of serum IL-18 had a positive correlation with the progression of severe hepatobiliary adverse effects related to the treatment with ceritinib and the involvement of IL-18 in the hepatobiliary inflammation by the pathological evaluation. These results suggest that IL-18 could be a useful surrogate marker for hepatobiliary toxicity of ceritinib. However, this is only one case report and further prospective observations will be needed to clarify this issue.

## Abbreviations

ALK: Anaplastic lymphoma kinase
CT: Computerized tomography
IL: Interleukin
MRI: Magnetic resonance imaging
NSCLC: Non-small cell lung cancer
